# Strength prediction of ECC-CES columns under eccentric compression using adaptive sampling and ML techniques

**DOI:** 10.1038/s41598-024-83666-z

**Published:** 2025-01-07

**Authors:** Khaled Megahed

**Affiliations:** Department of Structural Engineering, Mansoura University, PO BOX 35516, Mansoura, Egypt

**Keywords:** Machine learning, Engineered cementitious composites, Adaptive sampling, Finite element modeling, CatBoost model, Eccentric compression, Civil engineering, Structural materials, Computer science, Scientific data, Statistics

## Abstract

A novel type of concrete-encased steel (CES) composite column implementing Engineered Cementitious Composites (ECC) confinement (ECC-CES) has recently been introduced, offering significantly enhanced failure behavior, ductility, and toughness when compared to conventional CES columns. This study presents an innovative method for predicting the eccentric compressive capacity of ECC-CES columns, utilizing adaptive sampling and machine learning (ML) techniques. Initially, the research introduces a finite element (FE) model for ECC-CES columns, incorporating material and geometric nonlinearities to capture the inelastic behavior of both ECC and steel through appropriate constitutive material laws. The FE model was validated against experimental data, demonstrating strong predictive accuracy. An adaptive sampling process was employed to efficiently explore the design space, resulting in a database of 2,908 FE models. Subsequently, six machine learning models were used to predict the eccentric compressive capacity based on the generated FE database. These models were thoroughly evaluated and demonstrated superior prediction accuracy compared to established design standards like EC4 and AISC360. Based on evaluation metrics, the Gaussian Process Regression (GPR), CatBoost (CATB), and LightGBM (LGBM) models emerged as the most accurate and reliable, with over 97% of the finite element (FE) samples falling within a 10% error range. While the ML models demonstrate impressive performance, their black-box nature restricts their practical use in design applications. Consequently, this study introduces a proposed design that offers competitive performance metrics. The novelty of this work lies in integrating adaptive sampling through Bayesian Optimization (BO) with the power of machine learning (ML) to generate training data that effectively covers a large input space while minimizing error. SVR, CatBoost, and GPR models demonstrated mean μ, R^2^, and a20-index values near 1.0, with CoV and MAPE% values consistently low, indicating highly accurate predictions across testing subsets.

## Introduction

Concrete-encased steel composite (CES) columns are widely used in high-strength structural applications, including high-rise buildings and bridges, due to their remarkable strength, stiffness, and ductility. By combining the strength of steel with the durability of concrete, these columns achieve superior structural efficiency, enabling more compact and lightweight cross-sections compared to traditional reinforced concrete (RC) columns^1^. Additionally, the concrete encasement offers enhanced fire and corrosion resistance while delaying local buckling in the steel section, ultimately extending the column’s lifespan^2,3^. While CES columns have traditionally been constructed using normal-strength concrete (NSC), recent research has shifted focus towards the use of high-strength concrete (HSC) to further enhance their performance^2^^–^^4^. Although HSC offers various benefits, such as high strength and stiffness, it introduces challenges like premature spalling and brittle failure under compression. These issues can negatively affect both the load-bearing capacity and durability of the columns, potentially reducing their overall structural performance^5,6^.

To address these challenges, a new approach has been introduced by encasing CES columns with Engineered Cementitious Composites (ECC). This innovation helps mitigate associated with HSC, such as concrete spalling and brittle failure^5^^–^^7^. ECC is a remarkably ductile material, displaying greater tensile (ε ≈ 0.4 – 3%) and compressive (ε ≈ 0.38 – 0.6%) ductility than conventional concrete^5,6,8^. This increased ductility enables ECC to effectively control cracking and spalling, especially under high compressive loads, thereby enhancing the compressive performance of ECC-concrete encased steel (ECC-CES) composite columns^5^^–^^7^.

Due to the novel nature of ECC-CES columns, literature on both numerical and analytical models for predicting their compressive behavior is scarce. To fill this gap, a finite element (FE) model has been created using ABAQUS^9^, designed to accurately forecast the compressive response of ECC-CES columns under eccentric compression. This FE model integrates detailed material properties for each column component, employs suitable mesh sizes, accounts for geometric imperfections, and includes contact interface properties to accurately capture the complex inelastic behavior of the composite columns. The developed FE model has been validated against existing experimental studies, demonstrating strong agreement in initial stiffness, ultimate strength, and load-deformation responses^8^. This validated model offers a reliable framework for further exploration of ECC-CES column behavior, aiding researchers in investigating various design configurations and material properties without the need for extensive experimental testing.

To improve the efficiency of finite element (FE) models in predicting ECC-CES column strength, surrogate models—also called meta-models—are implemented. Surrogate models provide simplified approximations of computationally intensive simulations while maintaining a high level of accuracy. However, conventional design of experiments (DoE) techniques like factorial design, central composite design, and Latin Hypercube Sampling (LHS)^10^ often fall short when handling high-dimensional or nonlinear problems, leading to inefficiencies and excessive computational demands. These limitations are especially problematic in the context of ECC-CES columns, where variations in material properties, dimensions, and loading conditions require a more nuanced approach to model development.

Common techniques for surrogate modeling, particularly well-suited to overcome these previous challenges, involve Kriging models (or Gaussian Process models)^11,12^, polynomial regression surfaces^13^, support vector regression (SVR)^14^, and neural networks^15^. These methods have proven effective in diverse applications, such as investigating the behavior of rockfill dams through polynomial chaos expansion and deep learning networks^16^. The Bayesian optimization is then implemented to iteratively refine the surrogate model by selecting new samples based on local optimization or global accuracy criteria. This method—also named adaptive sampling—has been shown to improve model precision with fewer samples^12,17^. This approach systematically explores the design space, yielding a comprehensive database with 1,040 finite element (FE) specimens for columns subjected to major bending and 1868 FE specimens under minor bending.

Bayesian optimization (BO), a form of adaptive sampling, is particularly well-suited to overcome these challenges. It refines surrogate models by iteratively selecting the most informative new samples based on local optimization and global accuracy criteria. This approach is advantageous for ECC-CES columns as it ensures that data collection focuses on the most uncertain or significant regions of the design space, minimizing errors in predictions while reducing the number of required simulations. This adaptive sampling method, coupled with the power of machine learning, creates a comprehensive database that captures the complexity of ECC-CES column behavior with minimal computational resources. As demonstrated in this work, the use of adaptive sampling and machine learning techniques facilitates the generation of data that not only covers a broad range of scenarios but also ensures high prediction accuracy, enhancing the reliability of strength assessments for ECC-CES columns.

The generated FE database through the adaptive sampling strategy can be implemented effectively with machine learning (ML) techniques to predict ECC-CES columns compressive strength under eccentric compression. ML has become an effective tool in solving complicated structural engineering challenges, introducing accurate predictions while significantly reducing the need for extensive experimental and FE resources^18^^–^^22^. Using the generated FE datasets, ML models decrease the dependency on additional FE simulations, thus accelerating the model development process. For example, Tran et al.^23^ collected a dataset consisting of 300 samples from uniaxial loading tests to train ML models for the axial strength prediction of square concrete-filled steel tube (CFST) columns with reliable results. Furthermore, Zarringol et al.^24^ used a more extensive database of 3091 CFST columns with diverse geometries, including rectangular and circular columns with and without eccentricity, to train ML models for predicting compressive behavior under various loading conditions. Shu et al.^25^developed a point cloud and machine learning-based method for automated recognition and measurement of reinforcement cages and corrugated pipes in concrete beams, achieving high accuracy in position and spacing detection to ensure construction quality. In addition, Hou and Zhou^26^ introduced a range of ML models to refine the predictive accuracy for both short and slender circular-shaped CFST columns. Wang et al.^27^ explored CFST composite supports for stabilizing roadway intersections with complex cross-sections and stress concentrations. They highlighted the portal support frame as the key component for structural optimization and improved performance. They implemented various ML techniques, such as artificial neural networks (ANN), Gaussian process regression (GPR), genetic algorithms, radial basis function neural networks (RBFNN), and multiple linear regression (MLR). These optimized models demonstrated high precision in predicting the compressive strength of CFST columns, highlighting the significant potential of ML in modeling complex structural behaviors.

## Finite element modelling

Accurate prediction of structural member behavior is highly dependent on the proper characterization of material properties^4^. To improve both strength and ductility, researchers have developed hybrid fiber ECCs by integrating low-modulus fibers, such as polyvinyl alcohol (PVA), with high-modulus fibers like steel in a high-strength matrix^6,7,28^. The inclusion of steel fibers significantly enhances the fire resistance of ECC while effectively reducing crack widths, which decreases permeability and improves long-term durability. As a result, steel-PVA hybrid fiber ECC exhibits superior crack resistance, improved damage control, greater compressive toughness, and enhanced energy dissipation compared to conventional concrete.

The compressive stress–strain behavior of steel-PVA hybrid fiber ECC is unique, demonstrating nonlinear characteristics during the pre-peak ascending phase due to strain hardening^28^. In the post-peak descending phase, the material exhibits a bilinear relationship, characterized by an initial sharp reduction in load-bearing capacity, followed by a more gradual decline^29,30^, as outlined in Eq. (1). This detailed material modeling is essential for finite element simulations, enabling precise replication of structural behavior, especially when analyzing ECC-CES composite columns.

1 $${f}_{c}=\left\{\begin{array}{l}\frac{{f}_{c}^{\prime}\beta \left(\frac{{\varepsilon }_{c}}{{\varepsilon }_{c}^{\prime}}\right)}{\beta -1+{\left(\frac{{\varepsilon }_{c}}{{\varepsilon }_{c}^{\prime}}\right)}^{\beta }}, \qquad 0\le \frac{{\varepsilon }_{c}}{{\varepsilon }_{c}^{\prime}}\le 1.0\\ 2.5\left(1.0-0.6\frac{{\varepsilon }_{c}}{{\varepsilon }_{c}^{\prime}}\right){f}_{c}^{\prime}, \qquad 1.0\le \frac{{\varepsilon }_{c}}{{\varepsilon }_{c}^{\prime}}\le 1.5\\ \frac{1}{35}\left(10.25-\frac{{\varepsilon }_{c}}{{\varepsilon }_{c}^{\prime}}\right){f}_{c}^{\prime}, \qquad 1.5\le \frac{{\varepsilon }_{c}}{{\varepsilon }_{c}^{\prime}}\le 5.0\end{array}\right.$$

where *f'*_*c*_ and *ε'*_*c*_ represent the compressive strength at the peak point and its corresponding strain, respectively. The shape of the stress–strain curve is governed by the factor *β*, which controls the curvature of the ascending portion of the curve, calculated as:

2 $$\beta =0.115{f}_{c}^{\prime}+1$$

The stress–strain model of steel material employed is elastic-perfectly plastic, as previous researches has shown that different steel models have a negligible impact on the results of numerical analyses^5,31,32^.

**Fig. 1:**
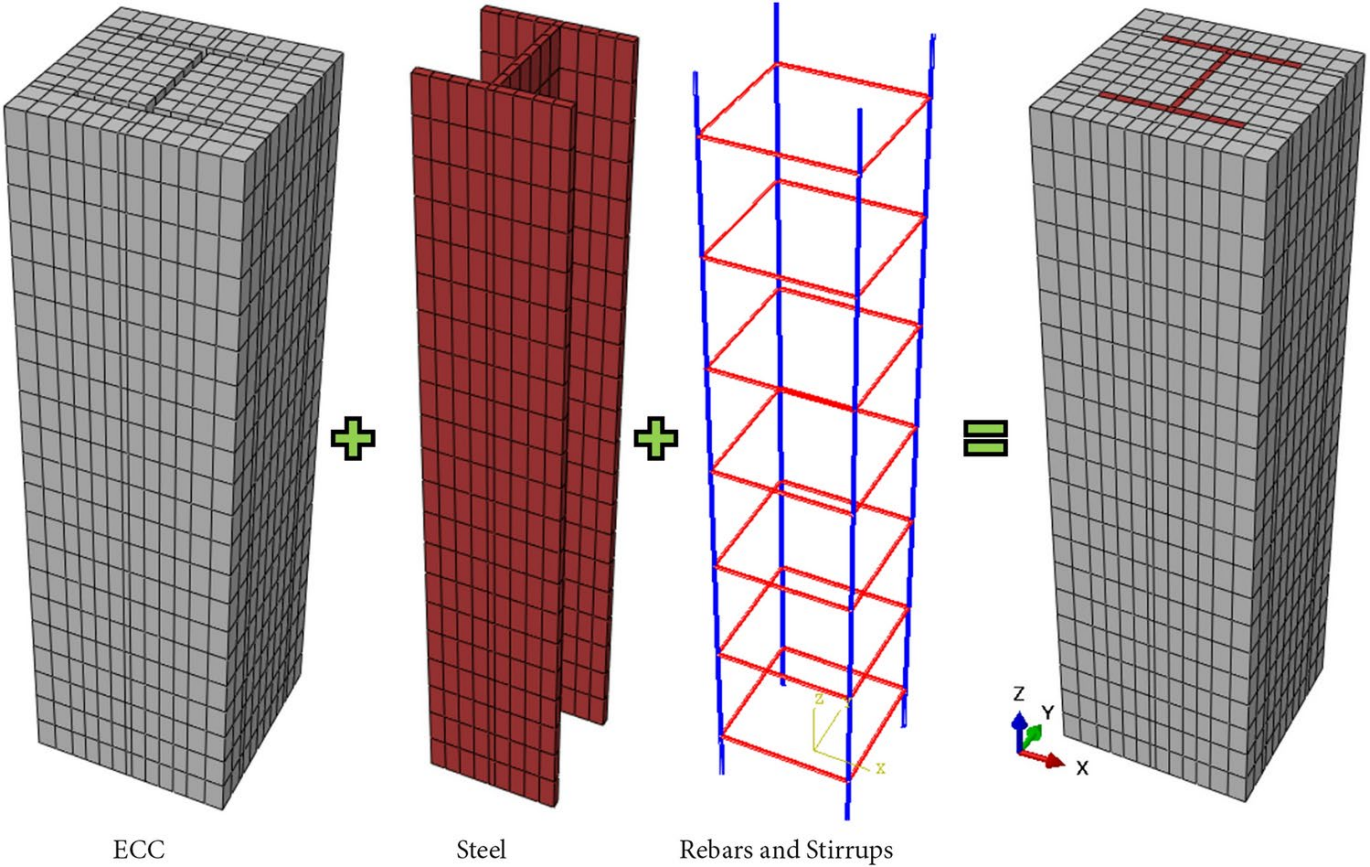
Geometric modeling of ECC-CES columns. The geometric modeling was captured from Abaqus version 6.14^9^.

To achieve accurate predictions, the proposed finite element (FE) model incorporated material and geometric nonlinearities, contact interactions, and geometric imperfections. For both the concrete and steel components, eight-node linear brick elements with reduced integration (C3D8R) from the ABAQUS element library were employed. These C3D8R elements, which feature three translational degrees of freedom per node, are widely used in nonlinear simulations involving contact between deformable bodies and plasticity under large deformations^9,32^. The transverse and longitudinal reinforcements were modeled with T3D2 truss elements—two-node elements embedded throughout the column. Figure 1 illustrates a typical configuration of the ECC-CES column elements. A static general analysis was conducted using a displacement control approach. The top and bottom loading areas were defined as rigid bodies with reference points, and the axial load was applied through displacement control at the top reference point. Lateral translational movements were constrained at the top of the column, allowing only axial deformation in the loading direction, while the bottom was fully fixed in all translational degrees of freedom.

To enhance prediction accuracy, the finite element (FE) model integrated material nonlinearities. For the ECC materials, the standard *ELASTIC option in ABAQUS was applied, while the Concrete Damaged Plasticity (CDP) model was employed to simulate concrete’s tensile and compressive behavior under low confinement pressures^5,9,32^. The CDP model is a continuum plasticity-based damage framework and requires five key parameters: dilation angle (*ψ*), flow potential eccentricity (e), the ratio of biaxial to uniaxial compressive strength (*f*_*b*_*/f’*_*c*_), the ratio of the second stress invariant on the tensile meridian to the compressive meridian (Kc), and the viscosity parameter (v). For ECC, these parameters were set to *ψ* = 30°, *e* = 0.1, *f*_*b*_*/f’*_*c*_ = 1.17, *K*_*c*_, = 0.7, and *v*= 0.001^32^. The steel and reinforcement materials were modeled using the *ELASTIC and *PLASTIC options from the ABAQUS material library, which defined the elastic-perfectly plastic behavior of these components.

## Verification of finite element model

**Fig. 2:**
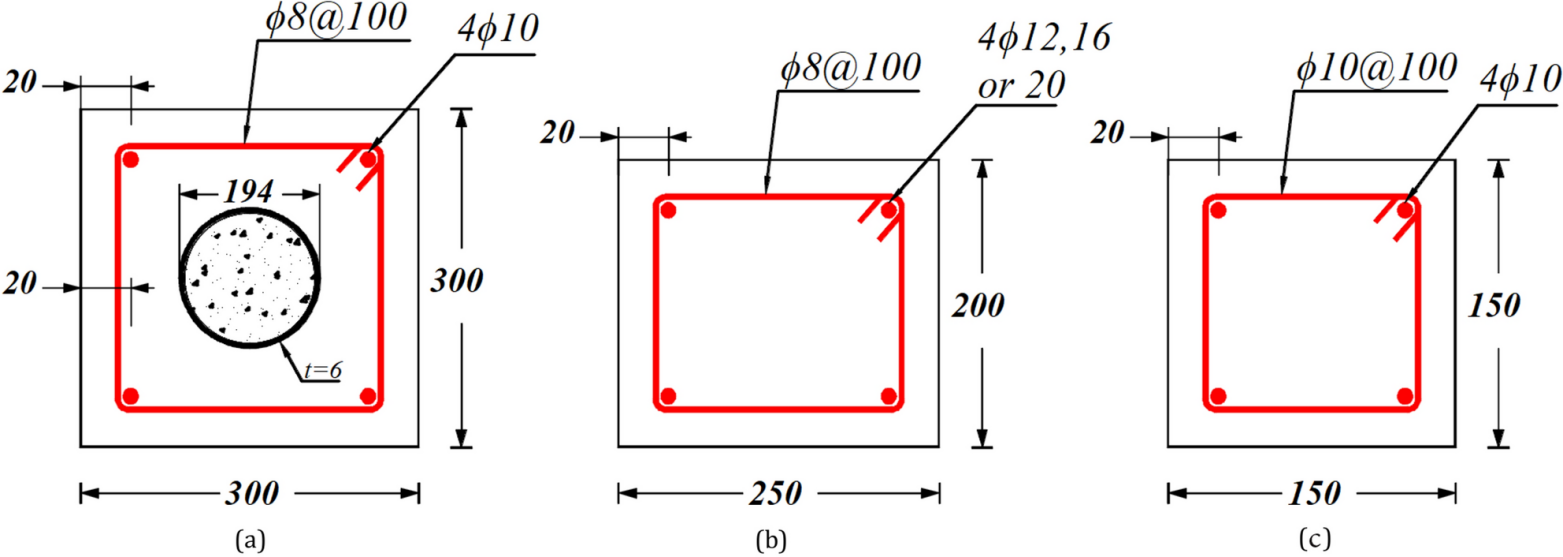
The cross-section geometry of the experiment test of CFS columns for verification. (**a**) C1-0.2(0.4,0.6,0.8), (**b**) E-12(16/20)−40(120), (**c**) EAe1(2)−0.5S-0.5P.

**Fig. 3:**
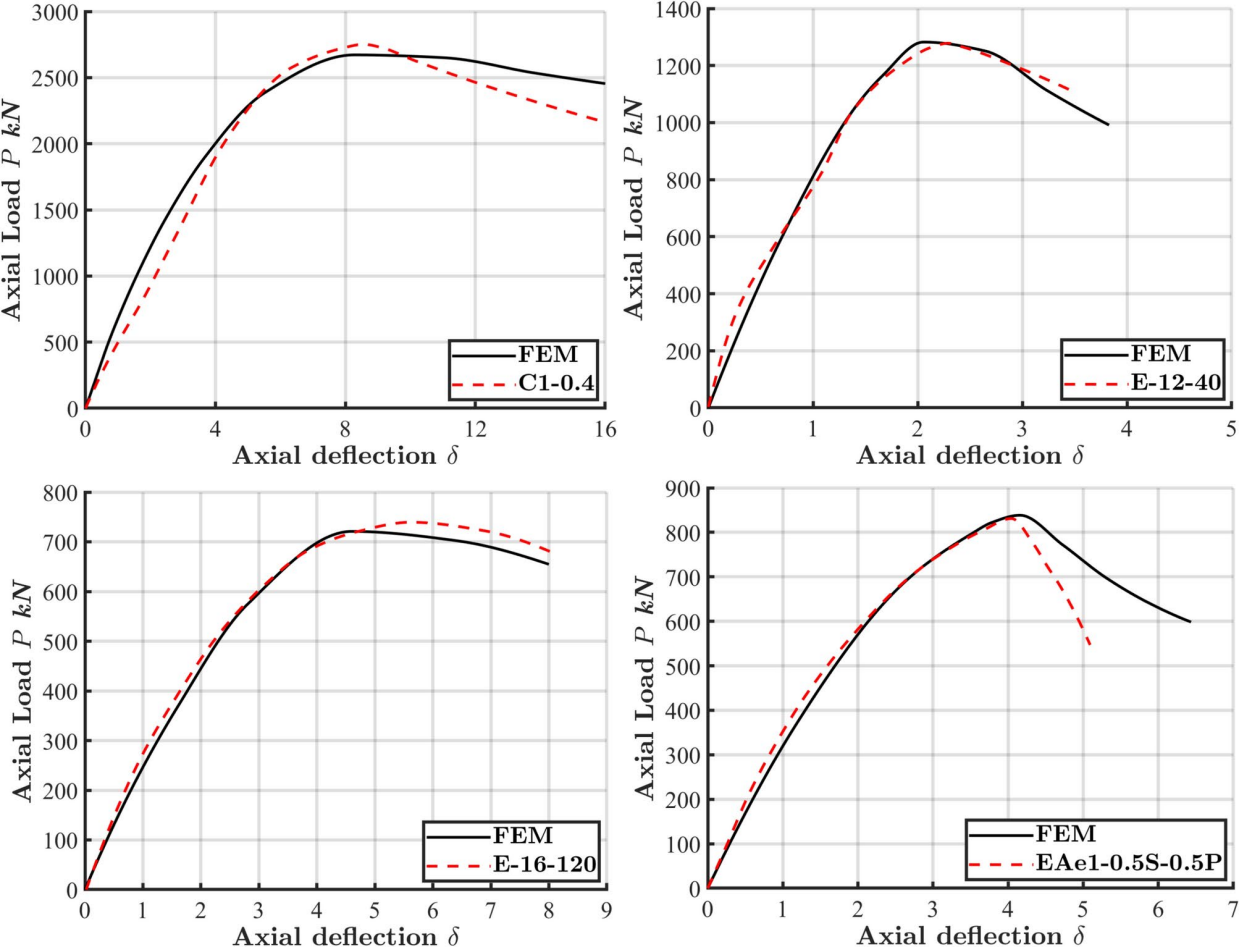
Comparison of load–deflection (L-D) response between experimental and numerical results for specimens C1-0.2^33^, E-12–40^34^, E-16–120^34^, and EAe1-0.5S-0.5P^35^.

The precision of FE model in predicting the ECC-confined concrete strength under eccentric compression was assessed by comparing numerical results with previous experimental tests, as detailed in Table 1 and illustrated in Fig. 2. Figure 3 displays the load–displacement (L-D) curves for four different column configurations: C1-0.2^33^, E-12–40^34^, E-16–120^34^, and EAe1-0.5S-0.5P^35^. The FE model showed strong reliability in predicting essential structural behaviors, including initial stiffness, peak strength, deformation, and post-peak responses. The L-D curves exhibit a linear behavior up to about 70% of the peak load, followed by a significant drop in load-carrying capacity. This sharp decline in strength was more pronounced in columns with higher-strength materials and low eccentricity due to the brittle behavior of these concrete.

**Table 1:** Specimens used for FE validation and comparison of FE analysis results with experimental results.

Specimen	$e$ (mm)	Figure [Ref]	Length (L)	Material strength			${\rho }_{l}$%*	${P}_{exp}$ (kN)	${P}_{FEM}$ (kN)	${P}_{FEM}/{P}_{exp}$
				${f}_{c}{\prime}$	${f}_{y}$	${f}_{r}$				
C1-0.2	60	Figure 2(a)^33^	1400	32.5	362	358	1.0	3671	3664	0.998
C1-0.4	120						1.0	2768	2752	0.994
C1-0.6	180						1.0	1846	1840	0.997
C1-0.8	240						1.0	1178	1161	0.986
E-12–40	40	Figure 2(b)^34^	1200	46.08	$-$	534	0.9	1278	1282	1.003
E-16–40	40					527	1.6	1409	1412	1.002
E-20–40	40					506	2.5	1572	1569	0.998
E-12–120	120					534	0.9	618	600	0.971
E-16–120	120					527	1.6	739	721	0.976
E-20–120	120					506	2.5	896	870	0.971
EAe1-0.5S-0.5P	30	Figure 2(c)^35^	1200	53.9	$-$	480	1.4	830	838	1.01
EAe2-0.5S-0.5P	70						1.4	515	487	0.946
									Mean	0.988
									CoV	0.0178

*$\rho_l$ Stands for the longitudinal reinforcement ratio in Fig. 2(c).

Moreover, the FE model’s peak strength predictions were compared with experimental tests, as illustrated in Table 1. The FE/test ratios, which are close to 1.0, reflect a strong correlation between the numerical results and experimental data, particularly concerning column strength. This close agreement emphasizes the model’s accuracy and reliability in simulating the compressive behavior of ECC-CES columns under eccentric compression.

## Bayesian optimization for adaptive experimental design

Traditional Design of Experiments (DoE) methods, including factorial design, central composite design, and Latin Hypercube Sampling (LHS)^10^, have been commonly used to construct surrogate models. However, these methods often struggle with high uncertainty or nonlinearity, leading to inefficiencies and excessive computational demands, particularly in high-dimensional problems. For instance, factorial designs, with their fixed size of *kd* for *k* levels and *d*dimensions, become impractical for problems with numerous input parameters. In contrast, Bayesian optimization enhances adaptive sampling by iteratively refining the surrogate model based on local optimization or global accuracy criteria. This approach has demonstrated significant improvements in model precision with fewer samples^12,17^.

Bayesian Optimization (BO) comprises two fundamental components^12,17,36^: a Gaussian process (GP) and an acquisition function. The GP models the design space probabilistically, providing both the predicted mean ${\mu }_{n}\left({\varvec{x}}\right)$ and the uncertainty ${\sigma }_{n}\left({\varvec{x}}\right)$ at any given input point ***x***, based on a set of prior observations ${D}_{1:n}=\left\{\left({{\varvec{x}}}_{1},{y}_{1}\right), \left({{\varvec{x}}}_{2},{y}_{2}\right), \dots \left({{\varvec{x}}}_{n},{y}_{n}\right)\right\}$, where ${{\varvec{x}}}_{i}$ defines the input and ${y}_{i}$ is its corresponding output at iteration *i*. This probabilistic model enables the prediction of function values at new points and the estimation of uncertainty, which is crucial for the acquisition function to determine the next experiment. The acquisition function chooses the most next promising point for further exploration by considering both the predicted mean ${\mu }_{n}\left({\varvec{x}}\right)$ and the uncertainty ${\sigma }_{n}\left({\varvec{x}}\right)$.

The GP is defined by its mean function $m\left({\varvec{x}}\right)$ and covariance function $k\left({\varvec{x}},{{\varvec{x}}}^{\prime}\right)$. The covariance function, also known as the "kernel," controls the smoothness of the GP process. The GP is expressed as:

3 $$f\left({\varvec{x}}\right)\sim \mathcal{G}\mathcal{P}\left(m\left({\varvec{x}}\right),k\left({\varvec{x}},{{\varvec{x}}}^{\prime}\right)\right)$$

The kernel $k\left({\varvec{x}},{{\varvec{x}}}^{\prime}\right)$ measures the degree of similarity between points in the input space, implying that if two points ***x*** and ***x'*** are “close” in terms of their inputs, the corresponding outputs *y* and *y'* are expected to be similar. A commonly used kernel is the Gaussian kernel, also known as the Radial Basis Function (RBF) kernel, which is defined as:

4 $$k\left({\mathbf{x}}_{i},{\mathbf{x}}_{j}\right)=\text{exp}\left(-\frac{{\left({{\varvec{x}}}_{i}-{{\varvec{x}}}_{j}\right)}^{T}\left({{\varvec{x}}}_{i}-{{\varvec{x}}}_{j}\right)}{2{l}^{2}}\right)$$

The variable *l* defines the length scale, which controls how quickly the correlation between points decreases with increasing spacing distance between them. A smaller *l* results in a faster decrease in correlation.

In practical applications, observations often include a noise component to account for measurement errors or variability in the system. This noise is modeled as normally distributed $\epsilon \sim \mathcal{N}\left(0,{\sigma }_{noise}^{2}\right)$ with zero mean. Therefore, the observation model can be defined as:

5 $$y= f\left({\varvec{x}}\right)+\epsilon$$

The inclusion of noise in the GP model allows Bayesian Optimization (BO) to effectively handle real-world experimental settings, where observations are often imperfect.

Gaussian Process Regression, also known as "kriging," is employed to estimate the objective function $f\left(\cdot \right)$ at a new location ***x*** during iteration $n+1$. This prediction results is a normal distribution characterized by a mean ${\mu }_{n}\left({\varvec{x}}\right)$ and an uncertainty ${\sigma }_{n}\left({\varvec{x}}\right)$, given by:

6 $$P\left({f}_{n+1}|{D}_{1:n},{\varvec{x}}\right)=\mathcal{N}\left({\mu }_{n}\left({\varvec{x}}\right),{\sigma }_{n}\left({\varvec{x}}\right)\right)$$

where

7 $$\begin{aligned}&{\mu }_{n}\left({\varvec{x}}\right)={k}^{T}{\left[K+{\sigma }_{noise}^{2}I\right]}^{-1}{y}_{1:n},\\ & \quad\sigma_{n}(x)=k(x,x)-k^T[K+\sigma^{2}_{noise}I]^{-1}k\end {aligned}$$

In these equations, *k* is the covariance vector $\left[k\left({\varvec{x}},{{\varvec{x}}}_{1}\right),k\left({\varvec{x}},{{\varvec{x}}}_{2}\right),\dots ,k\left({\varvec{x}},{{\varvec{x}}}_{n}\right)\right]$, and *K* defines the covariance matrix given by:

8 $$K=\left[\begin{array}{c}k\left({{\varvec{x}}}_{1},{{\varvec{x}}}_{1}\right)\hspace{0.25em}\hspace{0.25em}\hspace{0.25em}\hspace{0.25em}\cdots \hspace{0.25em}\hspace{0.25em}\hspace{0.25em}\hspace{0.25em}k\left({{\varvec{x}}}_{1},{{\varvec{x}}}_{n}\right)\\ \vdots \hspace{0.25em}\hspace{0.25em}\hspace{0.25em}\hspace{0.25em}\ddots \hspace{0.25em}\hspace{0.25em}\hspace{0.25em}\hspace{0.25em}\vdots \\ k\left({{\varvec{x}}}_{n},{{\varvec{x}}}_{1}\right)\hspace{0.25em}\hspace{0.25em}\hspace{0.25em}\hspace{0.25em}\cdots \hspace{0.25em}\hspace{0.25em}\hspace{0.25em}\hspace{0.25em}k\left({{\varvec{x}}}_{n},{{\varvec{x}}}_{n}\right)\end{array}\right]$$

After completing the *k*^th^ iteration of Bayesian Optimization (BO), a training dataset ${D}_{k}=\left\{{{\varvec{X}}}_{k}, {{\varvec{y}}}_{k}\right\}$ with *k* samples is generated. Using this dataset, a GP model $\left({f}_{k},{\mu }_{k},{\sigma }_{k}^{2}\right)\left({\varvec{x}}\right)$ is established. The next step involves selecting the most promising point ${{\varvec{x}}}_{k+1}$ for a new finite element (FE) simulation to optimize the process. To minimize the number of simulations, ${{\varvec{x}}}_{k+1}$ is chosen based on the current GP model $\left({f}_{k},{\mu }_{k},{\sigma }_{k}^{2}\right)\left({\varvec{x}}\right)$ and the information from ${D}_{k}$. This is achieved by maximizing the acquisition function, which assesses the potential of each point in the parameter space to improve the best-observed solution. Specifically, ${{\varvec{x}}}_{k+1}$ is determined by:

9 $${{\varvec{x}}}_{k+1}={\text{argmax}}_{{\varvec{x}}} EI\left({\varvec{x}}\right) \quad \text{subjected to }\mathbf{x}\in \left[{\mathbf{x}}_{\text{l}},{\mathbf{x}}_{\text{u}}\right]$$

where $EI\left(.\right)$ represents the Expected Improvement (EI) acquisition function. The Expected Improvement, introduced by Jones et al.^11^, is a widely used acquisition function that quantifies the expected gain in objective function value over the best observed result, defined as:

10 $$EI\left({\varvec{x}}\right)=\left({f}_{min}-\mu \left({\varvec{x}}\right)\right)\Phi \left(\frac{{f}_{min}-\mu \left({\varvec{x}}\right)}{\sigma \left({\varvec{x}}\right)}\right)+\sigma \left({\varvec{x}}\right)\phi \left(\frac{{f}_{min}-\mu \left({\varvec{x}}\right)}{\sigma \left({\varvec{x}}\right)}\right)$$

where Φ(⋅) and *ϕ*(⋅) represent the cumulative distribution function and probability density function of the standard normal distribution, respectively, and ${f}_{\text{min}}$ denotes e minimum observed value among $\left[f\left({{\varvec{X}}}_{1}\right),\dots ,f\left({{\varvec{X}}}_{k}\right)\right]$. The first term in *EI*(***x***) function promotes exploitation by focusing on improving the best observed error function value so far ${f}_{\text{min}}$, while the second term supports exploration by incorporating the prediction uncertainty $\sigma \left({\varvec{x}}\right)$.

Given that acquisition functions primarily depend on the mean $\mu \left({\varvec{x}}\right)$ and uncertainty $\sigma \left({\varvec{x}}\right)$ of the GP model, they are computationally less expensive to evaluate compared to the FE model. This efficiency allows Bayesian Optimization (BO) to effectively prioritize simulations at the most promising points, thus optimizing resource use and minimizing the total number of required simulations. At each iteration, after selecting a new point ${{\varvec{x}}}_{k+1}$, both the GP model and the acquisition function are updated. This iterative refinement reduces uncertainty ${\sigma }^{2}\left({\varvec{x}}\right)$ across the input space, and the process continues until the uncertainty is sufficiently minimized.

**Fig. 4:**
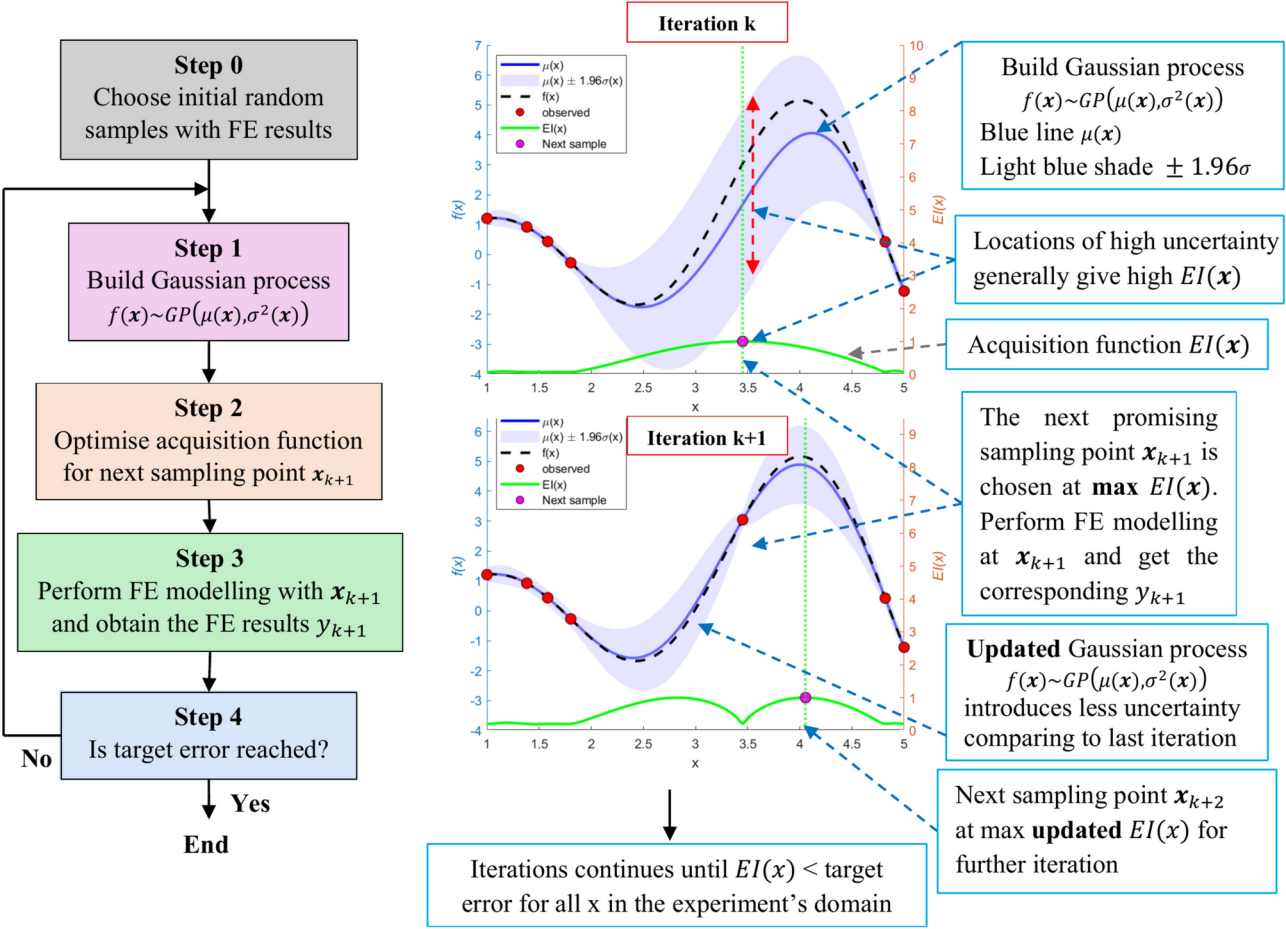
Flow charts of the adaptive sampling.

Figure 4 depicts the Bayesian optimization flowchart. On the right side, the figure presents a GP approximation of the objective function, with the acquisition function drawn below. In the GP model, $f\left(x\right)\sim GP\left(\mu \left({\varvec{x}}\right),{\sigma }^{2}\left({\varvec{x}}\right)\right)$, each data point ${{\varvec{x}}}_{i}$ is associated with a Gaussian distribution of function values characterized by a mean $\mu \left({{\varvec{x}}}_{i}\right)$ (solid blue line) and a variance ${\sigma }^{2}\left({{\varvec{x}}}_{i}\right)$ (light blue shaded region representing the 95% confidence interval). In the initial iterations, the surrogate model provides a relatively poor approximation of the true objective function (dashed line). The acquisition function effectively identifies the next evaluation point by balancing the trade-off between exploitation (areas with low mean) and exploration (areas with high variance). As new training data points are added, the GP model is progressively updated (refined), reducing variance and increasing accuracy in approximating the true function. This iterative process continues until a predefined target error, such as the root mean square error (RMSE), is achieved across the domain.

## FE database of ECC-CES columns

**Fig. 5:**
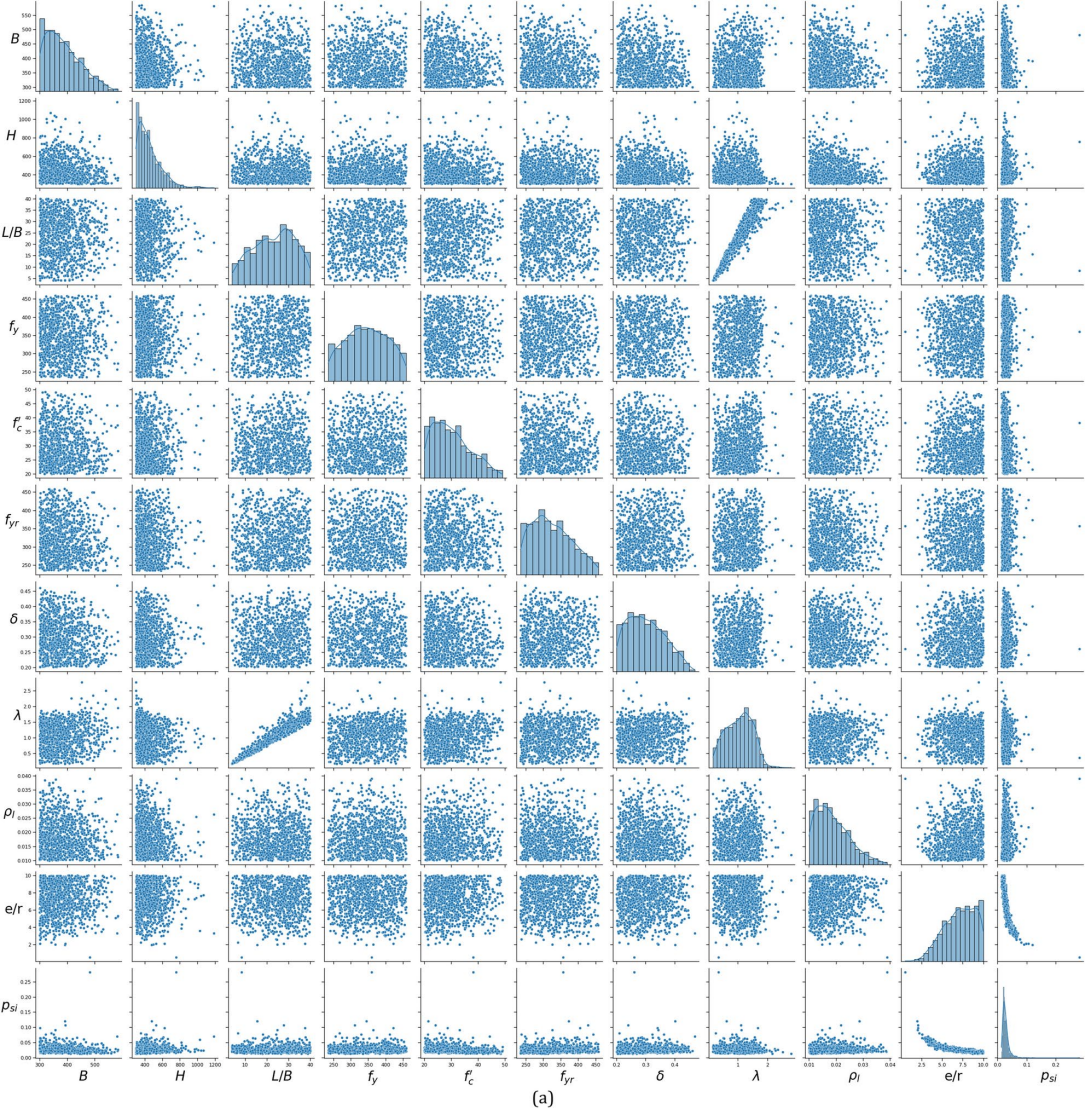

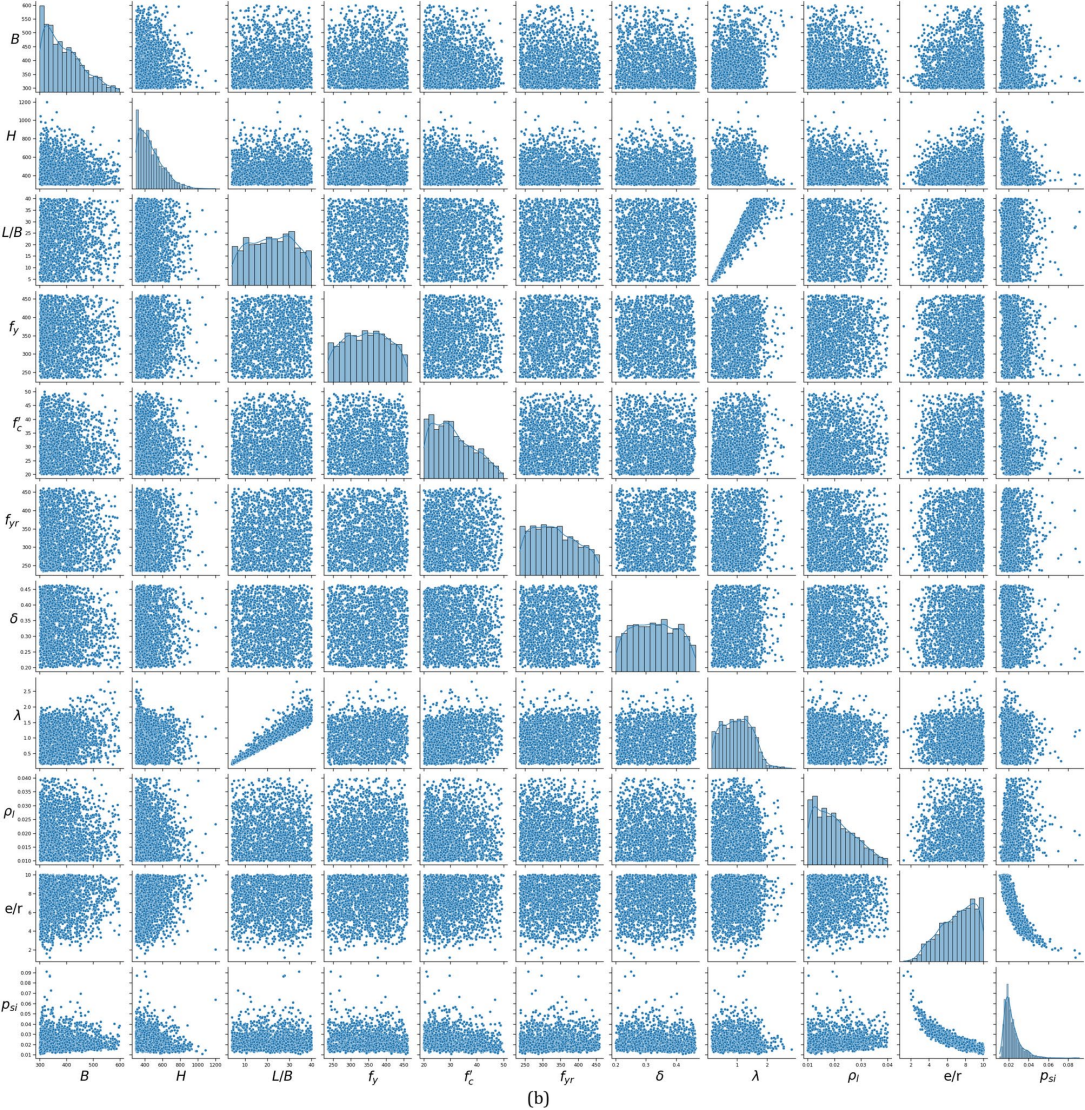
Statistical distribution of the database. (**a**) Major bending. (**b**) Minor bending.

The adaptive sampling strategy is utilized to systematically explore the design space, yielding a comprehensive database with 1,040 finite element (FE) specimens for columns subjected to major bending and 1868 FE specimens for columns under minor bending. Based on the results of different experimental and theoretical studies^5^^–^^8,33^^–^^35^, the capacity of ECC-CES columns under eccentric compression is influenced by several key components, which include the strength of concrete, steel, and longitudinal reinforcement. Therefore, 12 distinct design features were set as input variables, grouped into five categories: (1) concrete properties: column height (*H*), column width (*B*), Length-to-width ratio (*L/B*), and compressive strength (*f’*_*c*_); (2) steel section properties: steel section height (*h*_*s*_), flange width (*b*_*f*_), web thickness (*t*_*w*_), flange thickness (*t*_*f*_), and yield strength(*f*_*y*_); (4) longitudinal reinforcement properties: reinforcement ratio (*ρ*_*l*_), and yield strength (*f*_*yr*_); (5) load eccentricity (*e*). The output variable is the dimensionless strength index, denoted as *p*_*si*_. This index is calculated by normalizing the eccentric load *P*_*u*_ against the combined strengths of the column’s components, including the steel section, core concrete, and longitudinal reinforcement, defined as follows:

11 $${p}_{si}=\frac{{P}_{u}}{{N}_{pl}}, \quad {N}_{pl}={A}_{r}{f}_{yr}+{A}_{s}{f}_{y}+{A}_{c}{f}_{c}^{\prime}$$

where *A*_*s*_, *A*_*r*_ and *A*_*c*_ are the steel, longitudinal reinforcement, and concrete areas. Table 2 and Fig. 5 summarise statistical information for the output and the 12 input features from the established database under major and minor bending. The strength index, *p*_*si*_, reflects global column slenderness and load eccentricity effect, as a relatively slender column (characterized by a larger *L/B* ratio) with large load eccentricity results in a lower strength index value. It was found that using the strength index (*p*_*si*_) instead of column capacity (*P*_*u*_) as the main output enhances the ML prediction performance^37,38^.

**Table 2:** Statistic features of the FE dataset.

Variable	Symbol	Major bending						Minor bending					
		Min	Max	Mean	Std	Skewness	Kurtosis	Min	Max	Mean	Std	Skewness	Kurtosis
Column width	$B$(mm)	300	584	385	62	0.7	−0.18	300	600	390	70	0.77	−0.14
Column height	$H$(mm)	300	1200	452	130	1.48	3.08	300	1200	470	136	1.09	1.19
Length-to-width ratio	$L/B$	4	40	23.4	9.3	−0.2	−0.97	4	40	22	9.9	−0.03	−1.1
Reinforcement yield strength	${f}_{yr}$ (MPa)	235	460	325	56	0.39	−0.76	235	460	334	61	0.25	−0.99
Concrete strength	${f}_{c}^{\prime}$ (MPa)	20	50	30.3	7	0.59	−0.49	20	50	31	7.5	0.48	−0.73
Steel section yield strength	${f}_{y}$ (MPa)	235	460	346	59	−0.01	−0.99	235	460	345	61	0	−1.07
Steel contribution ratio	$\delta$*	0.2	0.47	0.3	0.06	0.34	−0.78	0.2	0.47	0.33	0.07	0.03	−1.12
Web thickness	${t}_{w}$ (mm)	1.7	40.6	6.8	3.1	2.23	14.64	1.8	27.1	8.5	4	1.16	1.49
Flange thickness	${t}_{f}$ (mm)	2.8	66.7	10.4	4.9	2.49	18.1	2.6	42.7	12.4	5.9	1.13	1.48
Steel section height	${h}_{s}$ (mm)	105	1092	307	136	1.39	2.97	108	1040	331	139	0.99	1.05
Flange width	${b}_{f}$ (mm)	104	479	242	70	0.53	−0.08	104	518	244	75	0.55	−0.15
Long. reinforcement ratio	${\rho }_{l}$	0.01	0.04	0.019	0.006	0.77	0.06	0.01	0.04	0.02	0.007	0.58	−0.54
Slenderness ratio	$\overline{\lambda }$**	0.16	2.78	1.05	0.44	0.03	−0.53	0.16	2.81	1	0.48	0.22	−0.58
Eccentricity ratio	$e/r$***	0.51	10	7.14	1.87	−0.42	−0.59	1.2	10	7.17	1.91	−0.47	−0.7
Axial load	${P}_{u}$(N)	49	6064	214	234	18.06	415	48	1683	193	91	3.92	44
Normalized load	${p}_{si}$****	0.013	0.281	0.028	0.013	8.02	130	0.011	0.091	0.023	0.009	2.11	8

*$\delta =\frac{{A}_{s}{f}_{y}}{{\text{A}}_{\text{s}}{f}_{y}+{A}_{r}{f}_{y}+{A}_{c}{f}_{c}^{\prime}}$ is the steel contribution ratio, where ${A}_{s}$, ${A}_{r}$, and ${A}_{c}$ are the steel, rebar, and concrete areas, respectively.

**Slenderness ratio $\overline{\lambda }$ is defined in Table 3for EC4^39^.

***$e/r$ is the eccentricity ratio with $r=H$ for major bending, $r=B$ for minor bending.

****${p}_{si}=\frac{{P}_{u}}{{\text{A}}_{\text{s}}{f}_{y}+{A}_{r}{f}_{y}+{A}_{c}{f}_{c}^{\prime}}$ is the normalized load.

The generated dataset in Table 2 ensures practical design adaptability by covering a broad range of parameters. For instance, the variations in column width (B) and height (H) span from 300 to 1200 mm and 300 mm to 1200 mm, respectively, while concrete strength (*f’*_*c*_) ranges from 20 to 50 MPa. Additionally, steel section yield strength (*f*_*y*_ ​) and reinforcement yield strength (*f*_*yr*_​) cover ranges from 235 to 460 MPa. Load scenarios, represented by the eccentricity ratio (e/r), vary from 0.51 to 10, allowing for exploration of different loading conditions. This comprehensive coverage ensures the dataset’s applicability for a variety of design scenarios and facilitates improved prediction models. It is important to acknowledge that the parameter ranges of CFST samples in the databases fall inside the scope of the EC4 design code^39^, as illustrated in Table 2 and Fig. 5.

## ML algorithms

This study utilizes six machine learning (ML) models to predict the eccentric strength of ECC-CES columns: categorical boosting (CatBoost)^40^, extreme gradient boosting (XGBoost)^41^, light gradient-boosting machine (LightGBM)^42^, random forests (RF)^43^, Gaussian Process Regression (GPR)^44^, and support vector regression (SVR)^45^. The performance of these models is evaluated and compared. Typically, ensemble learning methods provide higher accuracy and stability compared to individual models^40^.

CatBoost, LightGBM, and XGBoost are ensemble techniques based on boosting, where weak learners are combined iteratively to create a more robust predictor^46^. CatBoost is highly efficient for categorical features, eliminating the need for preprocessing non-numerical data^40^. It utilizes unbiased boosting to reduce gradient bias and enhance generalization, especially when handling categorical variables. LightGBM^42^uses a histogram-based approach for data splitting, making it faster and well-suited for large datasets. XGBoost^41^, by contrast, uses a level-wise depth-first strategy, which can be slower than LightGBM but may yield more robust results for specific tasks. Random Forests, introduced by Breiman^43^, is an ensemble learning method based on bagging. It trains multiple decision trees on different data subsets and combines their outputs through averaging (for regression) or voting (for classification). Key parameters influencing RF performance include the number of trees, maximum features, and tree depth.

## Proposed design

**Fig. 6:**
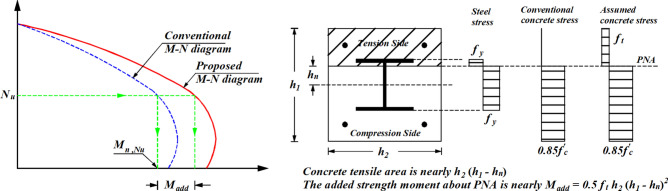
The proposed M–N interaction diagram (left), alongside the stress distribution, which compares conventional concrete stress with the assumed ECC concrete stress distribution that includes tensile strength *f*_*t*_ (right), with PNA denoting the plastic neutral axis.

Typically, the nominal flexural strength *M*_*n,Nu*_, corresponding to an axial load *N*_*u*_, is calculated assuming a rigid-plastic stress distribution within the composite section. This approach considers concrete’s compression strength at *0.85f*_*c*_*’* and neglects its tensile strength, while steel and rebar are assumed to reach yield strength on both the tension and compression sides, as shown in Fig. 6.

To consider the tensile strength of ECC concrete, this study assumed a tensile strength for the concrete on the tension side of the section, which enhances the resisting moment with a negligible impact on axial strength, as shown in Fig. 6. Assuming the concrete tensile area is ${h}_{2}\left({h}_{1}-{h}_{n}\right)$, the additional moment due to this tensile strength can be calculated around the plastic neutral axis (PNA) as follows:

12 $${M}_{add}=0.5{f}_{t}{h}_{2}{\left({h}_{1}-{h}_{n}\right)}^{2}$$

where *f*_*t*_ is the tensile strength, given in Eq. (13), extracted as the best fit to the FE database results, *h*_*1*_ and *h*_*2*_ are the dimensions parallel and perpendicular to the eccentricity direction, respectively, and *h*_*n*_ is the PNA axis height from the column’s centroid.

13 $${f}_{t}=0.2{\left({f}_{c}^{\prime}\right)}^{0.75}$$

To consider the slenderness effects, the applied second-order moment^39,47^ is given as follows:

14 $${M}_{u}=\left({N}_{u}e\right)\frac{1}{1-{N}_{u}/{N}_{cr}}, \quad {N}_{cr}=\frac{{\pi }^{2}{EI}_{eff}}{{\left(KL\right)}^{2}}$$

where $KL$ is the buckling length, and ${EI}_{eff}$ is the effective stiffness extracted as the best fit to the FE database results as follows:

15 $${EI}_{eff}={E}_{s}{I}_{s}+{E}_{s}{I}_{r}+0.9{E}_{c}{I}_{c}$$

where *E*_*s*_ and *E*_*c*_ are the Young’s modulus of steel and concrete materials, and *I*_*s*_, *I*_*r*_, and *I*_*c*_ represent the moments of inertia for steel, rebars, and the concrete part, respectively.

## Data preprocessing and hyperparameter Bayesian optimization technique

In this study, the min–max scaling technique is applied for data normalization to address challenges associated with multidimensionality. Following normalization, the dataset is split into two subsets: 80% is randomly allocated for training, while the remaining 20% is set aside for testing.

The performance of most machine learning (ML) algorithms is significantly influenced by their hyperparameters, which must be set prior to model training. Proper hyperparameter tuning is essential to achieve optimal predictive performance. Finding the best hyperparameters involves exploring various combinations and selecting the one that performs best on validation data. Traditional methods like grid search (GS) and random search (RS) can be exhaustive and time-consuming, particularly for models with numerous hyperparameters and large search spaces. In contrast, Bayesian Optimization (BO) uses surrogate functions, such as Gaussian processes and tree-structured Parzen estimators (TPE)^48^, to guide the search for the next hyperparameter set based on the performance of previous combinations. This approach reduces redundant evaluations, allowing BO to find the optimal hyperparameter configuration in fewer iterations compared to GS and RS methods^49^. For this study, the TPE model^39^was employed to optimize the hyperparameters of the ML models, owing to its superior robustness over other surrogate functions^49^.

## Performance and results of ML models

**Fig. 7:**
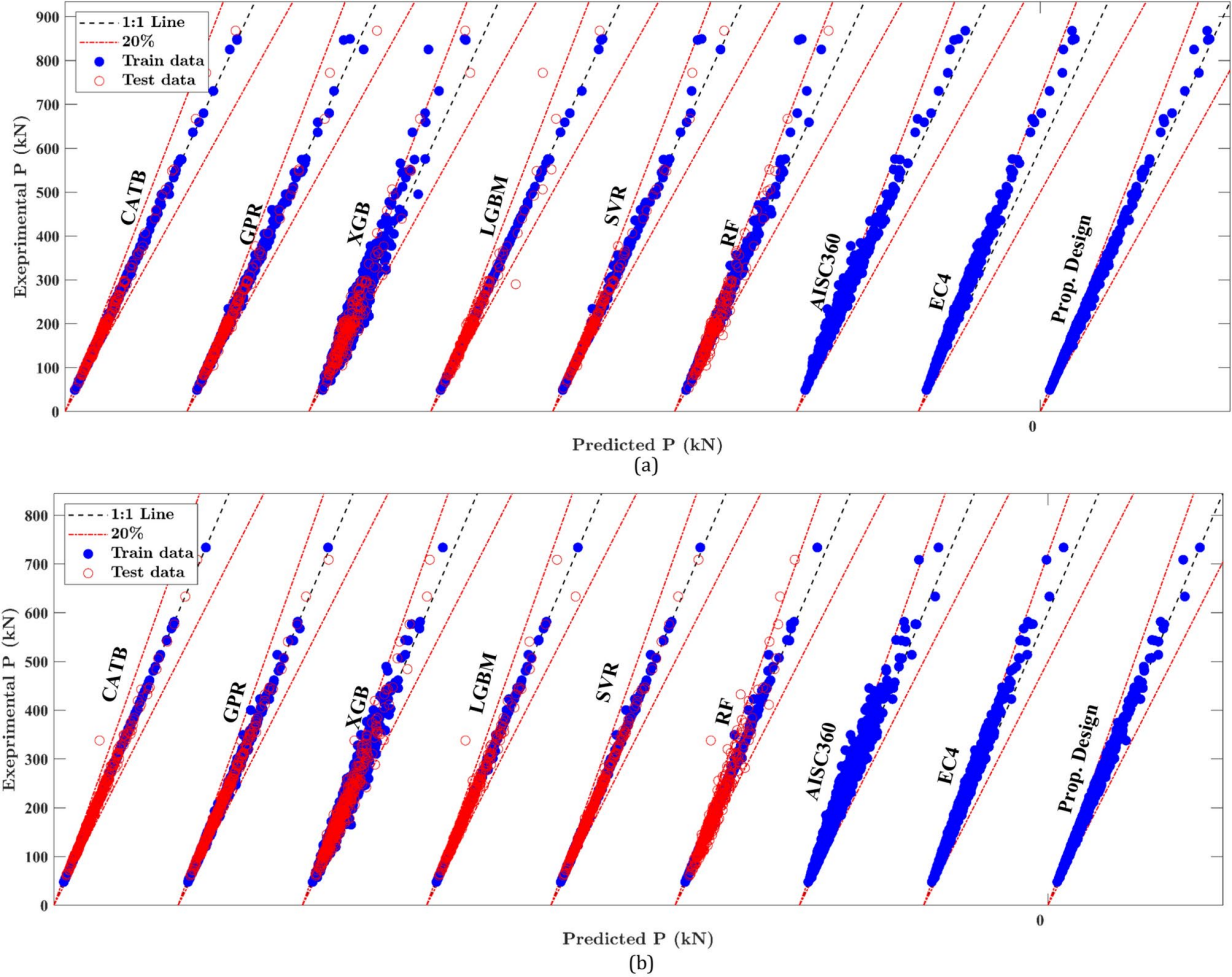
Prediction results of ML models, proposed design, and design standards. (**a**) Major bending. (**b**) Minor bending.

This section provides a detailed performance comparison of the machine learning (ML) models introduced in the study. The details of the developed ML models are provided in the supplementary data, including hyperparameter tuning processes and results. The scatter plots in Fig. 7 depict the relationship between FE and predicted strength for different ML models applied to the training and testing datasets for ECC-CES columns. In most cases, data points are tightly clustered around the diagonal line, demonstrating a strong correlation between model predictions and FE results. This strong alignment highlights the accuracy and reliability of the developed ML models.

Table 3 outlines several key evaluation metrics used to assess the performance of these ML models: (1) mean (*μ*), which defines the ratio between actual and predicted values, offering insight into overall model accuracy; (2) coefficient of variance (CoV), which reflects the variability of predictions relative to the mean, (3) coefficient of determination (*R*^*2*^), which indicates the proportion of variance in the dependent variable that is explained by the model, (4) root mean squared error (RMSE), which captures the average error in predictions, with a focus on more significant errors, (5) the mean absolute percentage error (MAPE), which evaluates the percentage error between actual and predicted values, and (6) a20-index^50^, which represents the percentage of predictions where the ratio of actual to predicted values lies between 0.80 and 1.20. The formulas for each of these metrics are provided as follows:

16 $$\mu =\frac{1}{n}\sum_{i=1}^{n}\frac{{y}_{i}}{{\widehat{y}}_{i}}, \quad {R}^{2}=1-\frac{\sum_{i=1}^{n}{\left({\widehat{y}}_{i}-{y}_{i}\right)}^{2}}{\sum_{i=1}^{n}{\left({y}_{i}-\overline{y }\right)}^{2}} \quad RMSE=\sqrt{\frac{1}{n}\sum_{i=1}^{n}{\left({\widehat{y}}_{i}-{y}_{i}\right)}^{2}}, \quad MAPE=\frac{1}{n}\sum_{i=1}^{n}\left|\frac{{y}_{i}}{{\widehat{y}}_{i}}-1\right|\times 100\%$$

where ${y}_{i}$ and ${\widehat{y}}_{i}$ are the actual FE and predicted strength values of the *i-*th sample, respectively, $\overline{y }$ is the mean value of FE output results, and *n* is the number of FE specimens in the database.

**Table 3:** Comparison of the developed ML models in predicting the strength of ECC-CES columns under eccentric compression.

Metrics	Training data			Testing data			All data					
	SVR	CatB	GPR	SVR	CatB	GPR	SVR	CatB	GPR	EC4^39^	AISC^47^	Prop. Design
*Major bending*												
Mean$\mu$	1.001	1	1	1	1	0.992	1.001	1	0.998	1.199	1.122	1.07
CoV	0.019	0.016	0.003	0.026	0.027	0.055	0.021	0.019	0.025	0.039	0.061	0.024
R^2^	0.997	1	1	0.999	0.997	0.98	0.997	0.999	0.996	0.951	0.956	0.961
MAPE%	0.85	1.313	0.226	1.651	2.096	3.703	1.01	1.469	0.922	19.929	12.324	7.097
RMSE(kN)	9.3	3.8	1.1	8.4	8.2	20.3	9.1	5	9.1	92	101.8	88.6
a20-index	1	1	1	1	1	0.99	1	1	0.998	0.501	0.88	0.999
*Minor bending*												
Mean$\mu$	1	1	1.006	1.001	1	1.004	1	1	1.006	1.135	1.076	1.045
CoV	0.009	0.004	0.005	0.017	0.035	0.051	0.011	0.016	0.023	0.041	0.067	0.028
R^2^	0.999	1	1	0.998	0.992	0.982	0.999	0.998	0.996	0.991	0.973	0.995
MAPE%	0.481	0.28	0.652	1.175	1.799	2.504	0.62	0.584	1.023	13.489	8.169	4.799
RMSE(kN)	2.2	0.7	2.1	3.8	7.9	11.9	2.6	3.6	5.7	25.5	20.7	10.6
a20-index	1	1	1	1	0.997	0.997	1	0.999	0.999	0.91	0.94	1

The evaluation metrics presented in Table 3 indicate that all machine learning (ML) models exhibit robust performance across both major and minor bending scenarios. For both major and minor bending columns, the mean *μ*, *R*^*2*^, and a20-index values for SVR, CatBoost (CatB), and GPR models are close to 1.0, along with small values for CoV, MAPE%, and RMSE values. The prediction results of these models exhibit CoV less than 0.06 and MAPE% lower than 3.7% for the testing subset, indicating minimized scattering in the prediction results compared to the experimental results. Specifically, the SVR model demonstrates the best performance for major and minor bending columns, with MAPE% values of 0.85 and 0.48 for the training set, and 1.65 and 1.18 for the testing set, respectively. Meanwhile, the CatBoost model shows MAPE% values of 1.31 and 0.28 for the training set, and 2.10 and 1.80 for the testing set, for major and minor bending columns, respectively. The GPR model also performs well with MAPE% values of 0.23 and 0.65 for training and 3.7 and 2.5 for testing, indicating the high accuracy of these models. Although the GPR model for major bending columns exhibits slightly higher errors for the testing data, achieving a MAPE% of 3.7 compared to the training set error with a MAPE% of 0.23, its overall performance, as measured by remaining evolution metrics, is comparable to other ML models. Additionally, for major bending columns, the SVR model achieves *μ* values of 1.0001 and 1.0, *R*^*2*^ values of 0.997 and 0.999, and a20-index values of 1.00 and 1.00 for the training and testing sets, respectively, all of which are near 1.00. Comparable performance is observed for minor bending columns as well. Such evaluation metrics reveal that the SVR model introduces the best prediction accuracy and predictive balance between the training and testing sets.

For minor bending columns, the metrics again reflect excellent predictive accuracy. The GPR model achieves a MAPE% of 0.65 for training and 1.02 for testing, while the CatB and SVR models yield MAPE% values of 0.28 and 0.48 for training, and 1.8 and 1.18 for testing, respectively. All models demonstrate a CoV below 0.05, suggesting minimal scattering in the predictions compared to experimental results.

When comparing the ML models with the proposed design, the proposed design shows performance metrics with a mean *μ* of 1.07 and a CoV of 0.024 for major bending and a mean *μ* of 1.045 and a CoV of 0.028 for minor bending. While the proposed design’s accuracy is slightly lower than that of the SVR and CatBoost models, it delivers results comparable to the remaining ML models. Moreover, it offers significant interpretability, making it more suitable for practical applications compared to the black-box nature of many ML models.

The proposed design predictions of ECC-CES columns were compared with the existing design code formulas, including EC4^39^and AISC360^47^. Although no specific design standards exist for ECC-CES columns, numerous researchers have observed that code-based predictions tend to be conservative, often underestimating the actual strength when compared to experimental results^5^^–^^8,33^^–^^35^. Table 3 shows that the proposed design achieves excellent performance, with a mean, *R*^*2*^, and a20-index close to 1.0, along with CoV values of 0.024 and 0.028, and MAPE% of 7.10% and 4.80% for major and minor bending columns, respectively. In contrast, the EC4 and AISC360 code standard for major bending columns exhibit significantly larger errors, with CoV values of 0.039 and 0.061 and MAPE% nearing 20% and 12.3%, respectively. For minor bending columns, the EC4 and AISC360 code standards present CoV values of 0.041 and 0.067 and MAPE% of approximately 13.5% and 8.2%, respectively. Additionally, compared to the proposed design, the AISC360 and EC4 predictions tend to significantly underestimate the eccentric capacity, particularly for major bending columns, as indicated by their low a20-index values and high mean values. This is further supported by the scatter plots in Fig. 7, where both AISC360 and EC4 display over-diagonal skewed distributions, highlighting their conservative approach to predicting eccentric capacity. While these design standards ensure safety (mean values larger than 1.0), the evaluation metrics of the proposed design reveal significantly lower error indices compared to the conventional standards, making it a more reliable and practical tool for accurately predicting column behavior under eccentric compression.

**Fig. 8:**
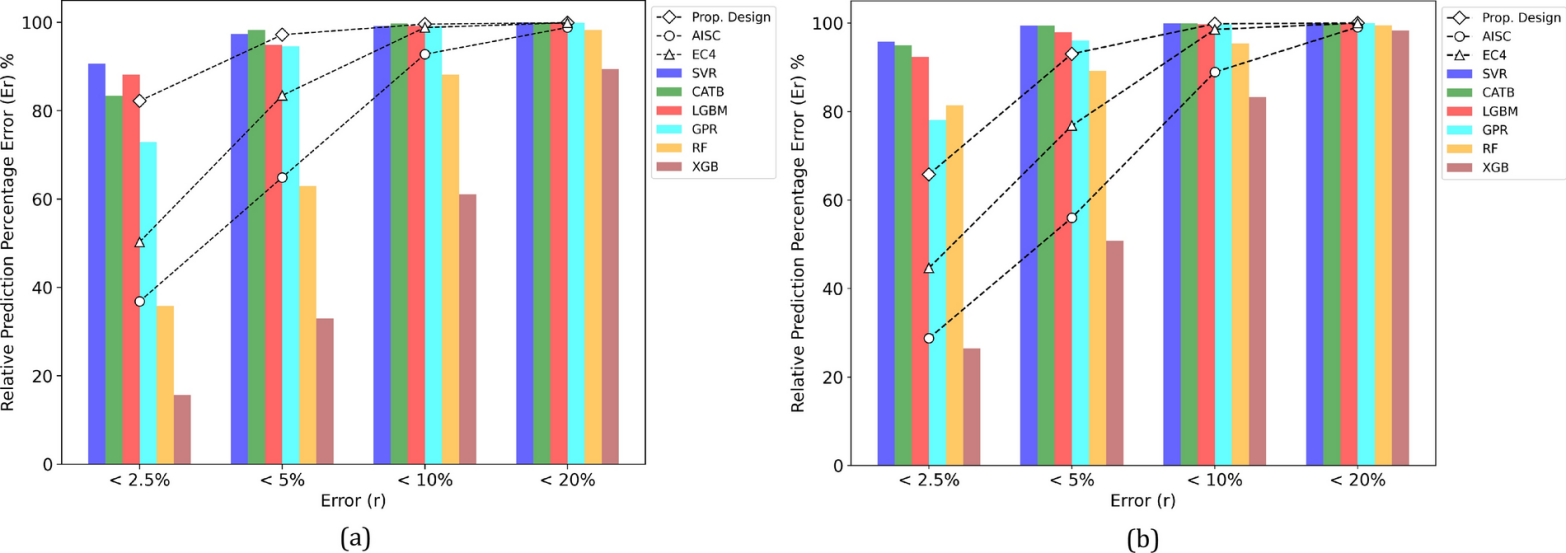
Prediction errors of design standards and established ML models. (**a**) Major bending. (**b**) Minor bending.

Figure 8 shows that most of the introduced ML models surpass traditional design standards in terms of accuracy, particularly within the 5% error range. The SVR, CATB, LightGBM (LGBM), and GPR models capture more than 94% of finite element (FE) samples within the 5% error range for major bending, whereas the proposed design, EC4, and AISC360 achieve 97.2%, 83.5%, and 64.9%, respectively. For minor bending, the SVR, GPR, CATB, and LGBM models also excel, with more than 96% of samples within the 5% error range, compared to 93% for the proposed design, 76.8% for EC4, and 56% for AISC360. These results demonstrate the superior predictive accuracy of the ML models, particularly GPR, CATB, and LGBM when compared to traditional design standards. Thus, the introduced ML models can be considered practical tools for estimating the eccentric capacity of ECC-CES columns with greater accuracy than conventional design standards. Furthermore, the proposed design outperforms EC4 and AISC360, showing nearly 1.5 times more samples within the 5% error range than AISC360 while delivering competitive results compared to the ML models.

## Limitations and future work

This section discusses the limitations of the developed data-driven models and identifies potential directions for future research. The validity of the introduced models is constrained within the range of minimum and maximum input parameter values specified in Table 2. These ranges define the applicability of the models and establish the boundaries within which accurate predictions can be made.

Machine learning (ML) is essential for advancing complex engineering analyses as it enables efficient data-driven modeling, optimization, and decision-making, which are crucial for enhancing accuracy and performance in various applications, including those involving advanced material processing and parameter optimization^18^^–^^22^. One of the main challenges in adopting machine learning (ML) models in engineering practice is their "black-box" nature, which often limits their interpretability and hinders their integration into practical design processes. While ML models such as SVR, CatBoost, and GPR provide highly accurate predictions of the eccentric capacity of ECC-CES columns, they do not directly offer insights into the physical behavior or mechanisms governing the structural response. To address this limitation, the proposed design combines the strengths of ML-based predictions with a framework rooted in well-established engineering principles, thereby providing interpretable and reliable solutions suitable for practical use.

The potential for future advancements in ECC-CES designs is vast, offering opportunities to enhance structural resilience and performance under diverse conditions. Insights from studies on FPB-isolated structures^51^highlight how ECC-CES designs can improve seismic resistance by enhancing stability and reducing pounding effects. Additionally, ECC’s superior tensile strength and crack resistance enable effective energy absorption in high-strain-rate impact scenarios^52^. Diao et al.^53^demonstrated the benefits of silicon carbide whiskers-modified mortars, suggesting their application to bolster ECC-CES resilience under dynamic loads through improved ductility and compressive strength. Comparative assessments of soil bagged graphite tailings suggest their potential as foundation materials, though challenges under high-eccentricity loading remain^54^. Advancements in 3D-printed cement paste using concave and convex trowels could inspire innovative ECC-CES fabrication methods to enhance interlayer bonding and structural integrity^55^. Huang et al.^56^ emphasized the role of axial compression and torsion-bending ratios in RC column performance, guiding reinforcement strategies for ECC-CES columns under complex loading. Guo et al.^57^ highlighted the load redistribution benefits of RC slabs and beams, paralleling ECC-CES columns’ capacity for superior energy dissipation and structural integrity during partial failures. Lastly, integrating precast systems with artificial controllable plastic hinge mechanisms, as studied by Huang et al.^58^, or viscoelastic materials, as shown by Zhang^59^, could provide ECC-CES columns with enhanced seismic resilience and vibration damping capabilities.

## Conclusion

This study developed a 3D nonlinear finite element model for analyzing ECC-CES columns under eccentric compression, incorporating both material and geometric nonlinearities. An adaptive sampling process was implemented to generate 2,908 finite element (FE) models, enabling efficient design space exploration. Additionally, six widely used machine learning (ML) models were employed, and their performance was rigorously evaluated and compared. The following conclusions can be drawn:

1. The FE model exhibited a high level of accuracy in predicting the compressive behavior of ECC-CES columns, with numerical results closely correlating with experimental data. The FE/test ratios consistently approached 1.0, validating the model’s reliability.
2. Adaptive sampling significantly enhances the accuracy of surrogate models used for predicting column strength by directing computational resources to areas of high uncertainty or significant impact. This method reduces the number of simulations required while ensuring comprehensive model performance across the entire input domain.
3. The developed machine learning models, particularly SVR, CATB, GPR, and LGBM, demonstrated superior predictive accuracy in estimating the eccentric capacity of ECC-CES columns compared to traditional design standards such as EC4 and AISC360.
4. Although black-box ML models can be challenging to interpret, the proposed design offers interpretable and practically valuable insights for engineering applications.
5. The proposed design outperformed existing code formulas, achieving nearly mean, *R*^*2*^, and a20-index values close to 1.0, low error metrics, establishing it as a strong competitor to both ML models and design codes in terms of accuracy and practical implementation.
6. Traditional design standards, such as EC4 and AISC360, were found to underestimate column capacity, resulting in higher mean errors and conservative predictions. These standards exhibited relatively large prediction errors (RMSE, MAPE) compared to the introduced ML models and the proposed design.
7. The introduced ML models developed through adaptive sampling and machine learning provide an efficient and accurate tool for estimating the eccentric capacity of ECC-CES columns under various loading conditions, reducing reliance on costly experimental testing.

The study highlights the potential of ML models to complement existing design standards. The superior accuracy of ML models, especially in scenarios where design codes tend to be conservative, indicates that ML can be a valuable tool for refining and validating design calculations in engineering practice.

## Data Availability

All data generated or analyzed during this study are included in this published article and available in a public repository https://github.com/kmegahed/eccentric-capacity-of-CFST-columns.
